# Epithelial–mesenchymal transition inhibition by metformin reduces melanoma lung metastasis in a murine model

**DOI:** 10.1038/s41598-022-22235-8

**Published:** 2022-10-22

**Authors:** Emerson Soares Veloso, Bárbara Andrade de Carvalho, Felipe Henrique de Souza Silva, Thaís Salviana Ribeiro, Bruna Mendes Lima, Camila Pereira Almeida, Vítor Henrique Soares Romão da Silva, Sara Aparecida Rocha, Marina Rios de Araújo Campos, Helen Lima Del Puerto, Enio Ferreira

**Affiliations:** Department of General Pathology, Institute of Biological Sciences, Universidade Federal de Minas Gerais, Belo Horizonte, Minas Gerais 31270-901 Brazil

**Keywords:** Cancer models, Melanoma, Metastasis

## Abstract

Melanoma is an aggressive cancer with fast metastatic spread and reduced survival time. One common event during the neoplastic progression is the epithelial–mesenchymal transition (EMT), which enhances invasiveness, cell migration, and metastasis. In this study, we investigated the effects of metformin at EMT in melanoma cell lines B16-F10 and A-375, in vitro, and the impact of EMT downregulation on melanoma progression in vivo. The metformin cells treatment reduces the migration potential in vitro and reduced the development of pulmonary metastases and the expressions of N-cadherin, vimentin, ZEB1, and ZEB2 at the metastases site, in vivo. These results indicate that metformin can promote EMT downregulation impairing the metastatic potential of melanoma cells.

## Introduction

Despite representing only 3% of cases of skin cancer, malignant melanoma presents significant clinical relevance by being recognized as one of the main causes of cancer death^1,2^. An important step during melanoma progression is the loss of interaction between melanocytes and keratinocytes, mediated by adhesion molecules such as E-cadherin^3^.

Epithelial–mesenchymal transition (EMT) is a process that allows cells to change between epithelial to mesenchymal phenotypes, which eventually promotes dedifferentiation and increases the capacity for cell invasion and migration. Initially reported in a physiological context during the embryonic period EMT has also been associated with neoplastic progression^4,5^. One of the changes promoted by EMT is the modification in cell adhesion mediated by cadherin switch, downregulated E-cadherin, and upregulated N-cadherin^6^. Although the melanocyte is not an epithelial cell, but neuroectodermal, there is evidence that EMT-like changes occur in melanoma and may be associated with neoplastic progression^7,8^.

EMT regulation is a complex process that involves the participation of transcriptional and translational regulators, such as EMT-inducing transcription factors (SNAIL, ZEB, and TWIST families) and miRNA200c (associated with the maintenance of epithelial differentiation)^9–11^.

Metformin, a normoglycemic agent, has been epidemiologically associated with a lower risk for cancer development and mortality^12,13^. Clinical trials and experimental studies have demonstrated the antineoplastic action of metformin and its ability to inhibit the formation of metastases in several neoplasms^14,15^. One of its effects is the inhibition of EMT with consequent reduction of motility, migration, and invasion of neoplastic cells, reported in vitro and in vivo including in melanomas^16,17^.

Despite the existing data on the role of EMT in cancer, its clear picture is yet to be elucidated in melanoma metastasis. Therefore, this study aimed to evaluate the effects of chemical inhibition of EMT with metformin on the expression of EMT-related markers and metastatic capacity in models of melanoma.

## Results

### Metformin affects cell viability in A-375 and B16-F10 cells in higher dose after a long-term exposure

**Figure 1:**
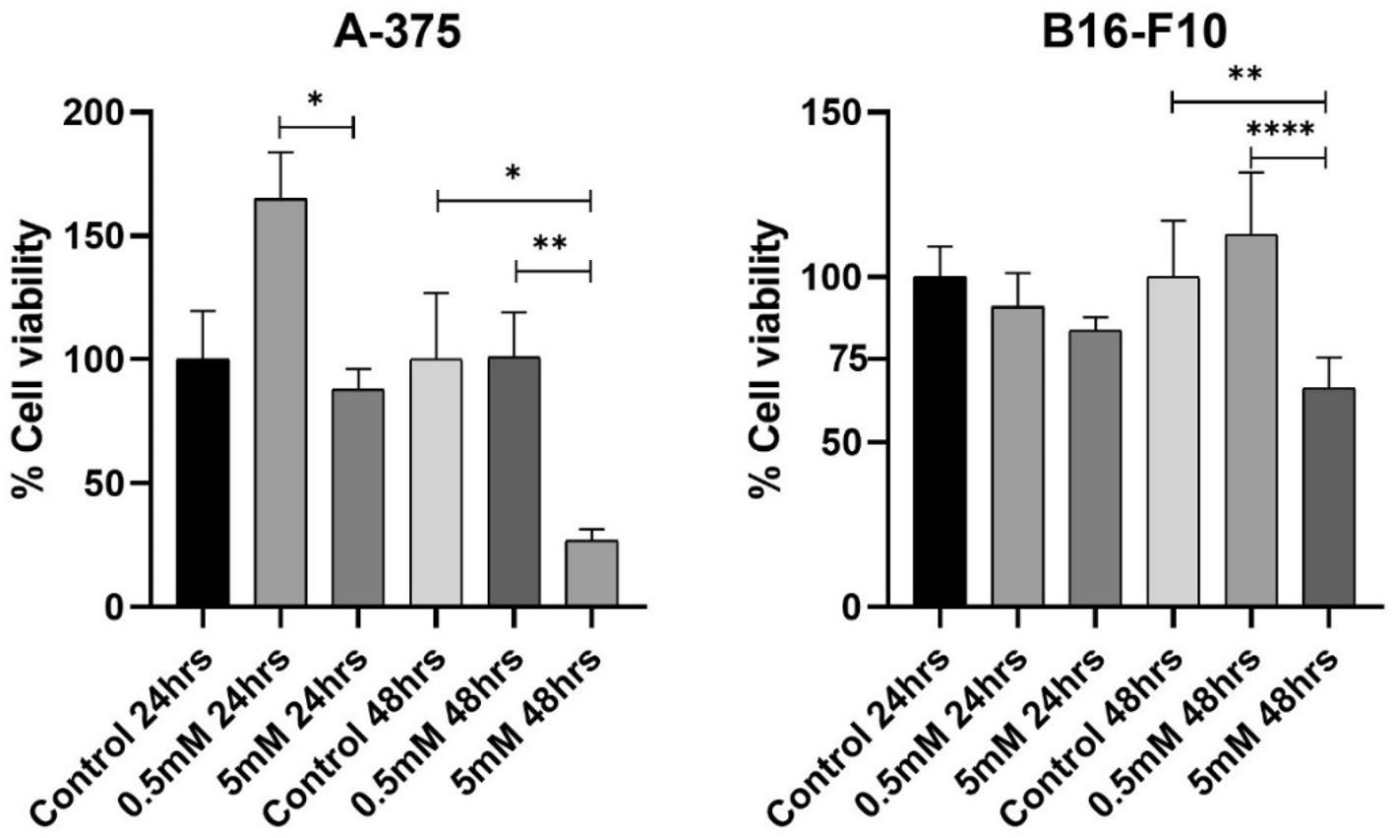
Effects of metformin on cell viability. Cells viability assessment by MTT assay of A-375 and B16-F10 cells lines treated for 24- and 48-h with metformin at 0.5 mM and 5 mM. *p < 0.05; **p < 0.01; ****p < 0.0001 (One-way ANOVA).

As shown in Fig. 1 the viability of 0.5 mM and 5 mM treated A-375 cells for 24 h did not differ from the non-treated cells, however, there was a reduction in cell viability in 5 mM treated cells compared to 0.5 mM treatment (p:0.0122). The 48-h treatment promoted the reduction of 5 mM treated cells viability compared to the non-treated (p:0.0172) and 0.5 mM treated cells (p:0.0065). The 24-h treatment did not reduce the cell viability of B16-F10 cells, but the 48-h treatment promoted the reduction of cell viability in the 5 mM treated cells compared to non-treated (p:0.0026) and 0.5 mM treated cells (p:0.0001).

### Metformin reduces A-375 and B16-F10 migration, without impairing EMT-related genes expression

**Figure 2:**
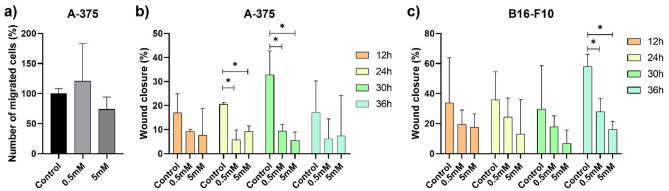
Effects of metformin on cell migration and invasion. (**a**) Number of A-375 invasion cells treated for 24-h with 0.5 mM or 5 mM metformin. (**b**,**c**) Wound closure percentual of A-375 (**b**) and B16-F10 (**c**) cells treated with 0.5 mM or 5 mM metformin. *p < 0.05 (One-way ANOVA).

The percentage of invasive A-375 cells was increased by treatment with 0.5 mM metformin (120%) and reduced with 5 mM treatment (73%), but without statistical significance (Fig. 2a). In the experiment conducted with B16-F10 cells, it was not possible to observe cell invasion under any of the conditions analyzed, including non-treated cells. Treatment with metformin, regardless of concentration, reduced the percentage of wound closure in A-375 (Figs. 2b, S1) and B16-F10 (Figs. 2c, S2) cells, especially with 24 and 30 h in A-375 cells and 36 h in B16-F10 cells.

**Figure 3:**
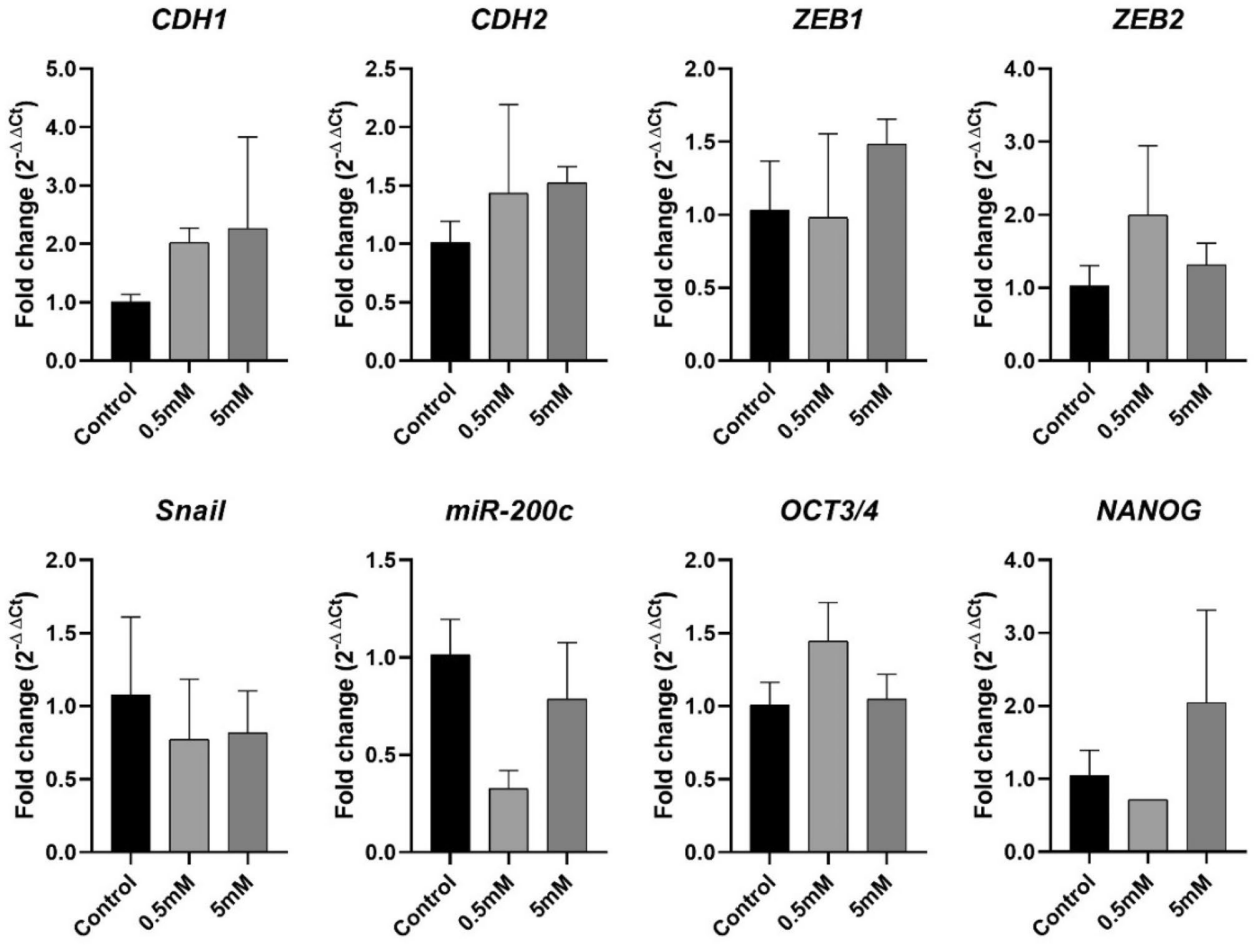
A-375 mRNA expression. A-375 cell line mRNA expression of EMT-related genes after 24 h of treatment with metformin at 0.5 mM and 5 mM. Data are plotted as mean ± SD of 2^−ΔΔCT^, directly proportional to the relative gene expression.

**Figure 4:**
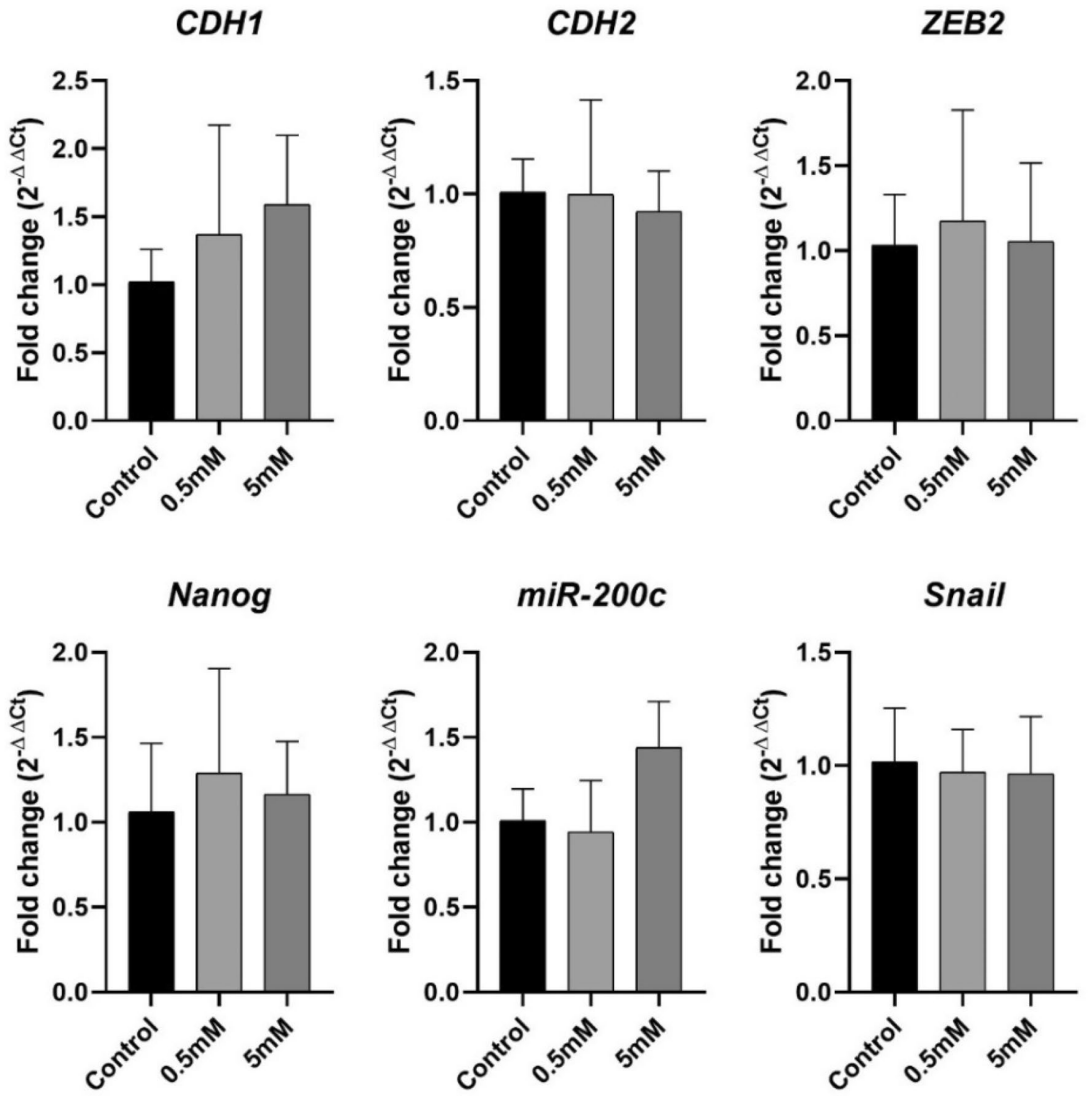
B16-F10 mRNA expression. B16-F10 cell line mRNA expression of EMT-related genes after 24 h of treatment with metformin at 0.5 mM and 5 mM. Data are plotted as mean ± SD of 2^−ΔΔCT^, directly proportional to the relative gene expression.

Metformin treatment did not significantly change the mRNA expression of all analyzed genes in A-375 and B16-F10 cells (Figs. 3 and 4). In A-375 cells the fold changes of *CDH1* expression were 2.02 and 2.26 in 0.5 mM and 5 mM treated cells, respectively. For *CDH2* the fold changes were 1.43 and 1.53, respectively. In B16-F10 cells the fold change of *CDH1* expression was 1.37 and 1.59 in 0.5 mM and 5 mM treated cells, respectively. For *CDH2* the fold changes were 0.99 and 0.92, respectively.

### Metformin reduces formation, size, and proliferation in the pulmonary metastasis of murine B16 melanoma

**Figure 5:**
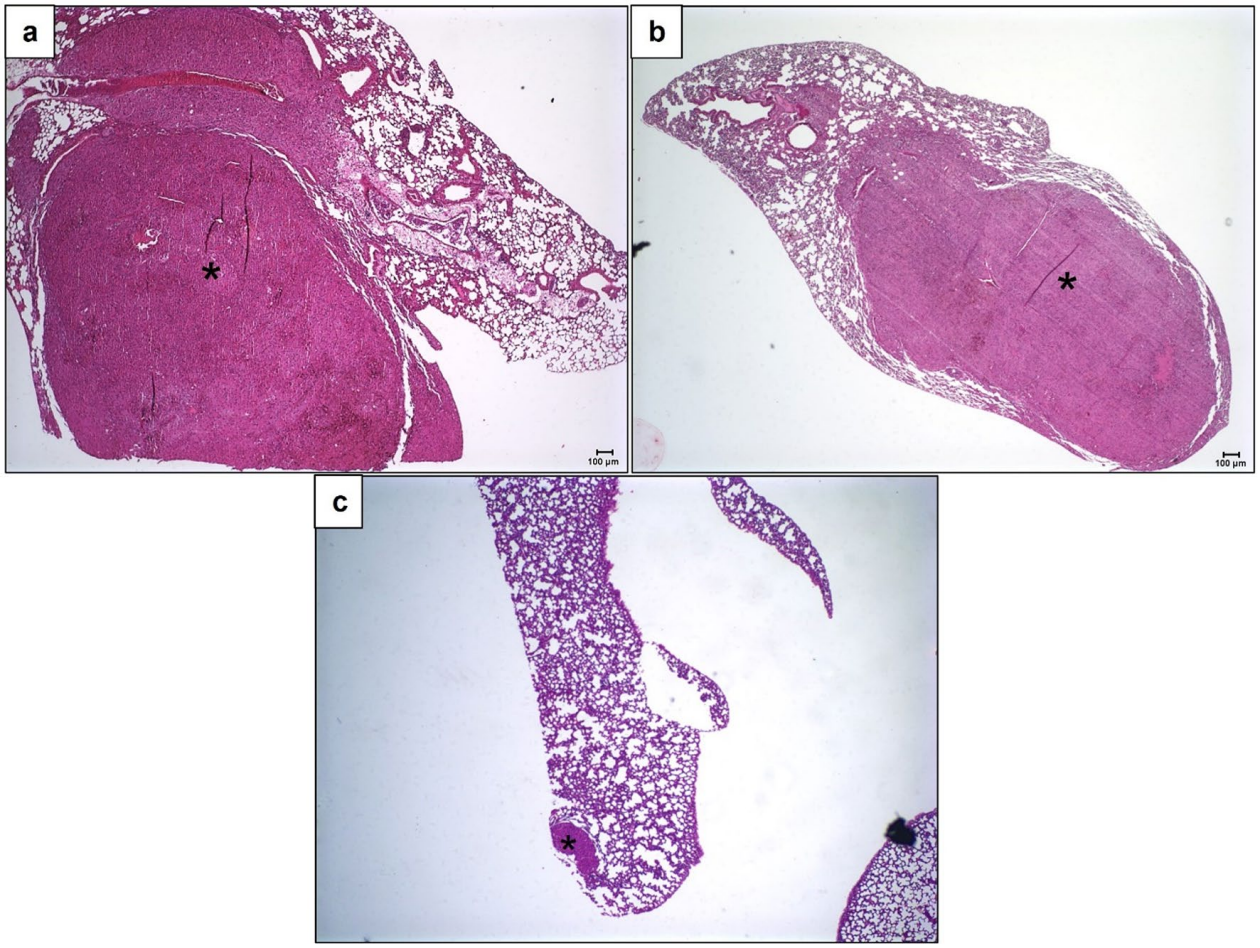
Lung metastases development in C57BL/6 mice inoculated with metformin-treated or untreated B16-F10 cells. Lung tissue, stained with hematoxylin–eosin, 21 days after inoculation of B16-F10 cells: (**a**) untreated with metformin; (**b**) treated, in vitro*,* with 0.5 mM of metformin; (**c**) treated, in vitro, with 5 mM of metformin. ×4. Scale bar: 100 µM.

**Figure 6:**
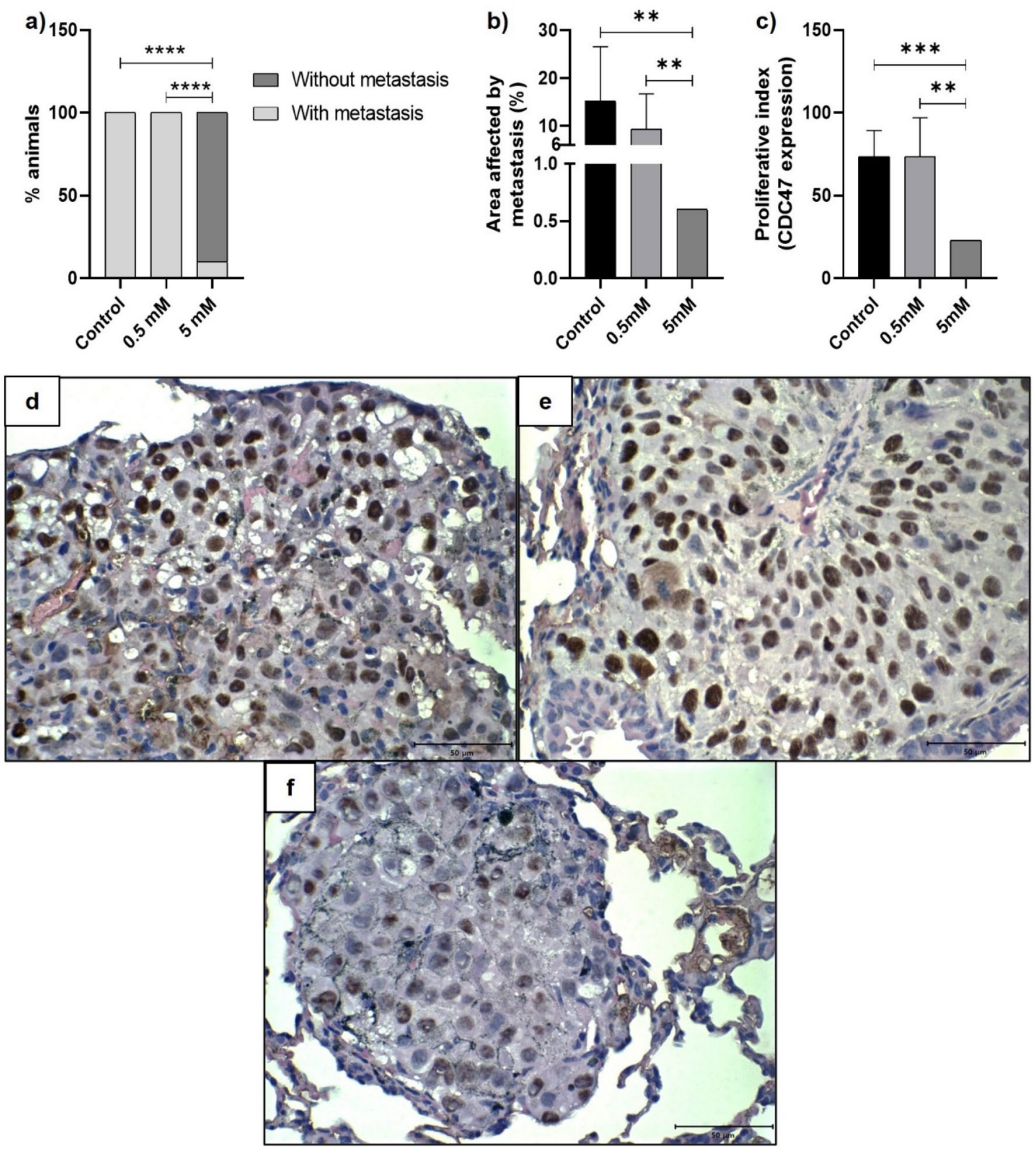
Histopathological and immunohistochemical parameters of pulmonary metastases from metformin-treated or untreated B16-F10 cells in C57BL/6 mice. (**a**) Frequency of pulmonary metastases. Fisher’s exact test. (**b**) Measurement of the pulmonary area affected by metastases. One-sample t-test. (**c**) Proliferative index of pulmonary metastases. One-sample t-test. **p < 0.01; ***p < 0.001; **** p < 0.0001. (**d**–**f**) Lung tissues, collected 21 days after inoculation of B16-F10 cells, were submitted to immunohistochemistry technique for CDC47 identification. Nuclear immunoexpression was evaluated in 500 neoplastic cells, allowing the determination of the mean proliferative index. (**d**) Cells untreated with metformin; (**e**) Cells treated, in vitro*,* with 0.5 mM of metformin; (**f**) Cells treated, in vitro, with 5 mM of metformin. ×60. Scale bar: 50 µM.

During the daily check-up of the inoculated mice, no clinical/behavioral changes were observed. Twenty-one days after inoculation all animals (control and that received 0.5 mM treated cells) presented pulmonary metastatic nodules, with extensive involvement of the lung parenchyma, associated with perivascular and peribronchiolar infiltrate of inflammatory cells (Fig. 5a,b). In the animals that received 5 mM treated cells, pulmonary nodules were observed in just one animal, organized in a small and rare focus of micrometastasis (Fig. 5c). The frequency of lung metastases was significantly lower in the 5 mM group than in the 0.5 mM and control and groups (Fisher's exact test, p < 0.0001, Fig. 6a).

The total lung and metastatic lung areas were used to determine the percentage of lung areas with metastases. The metastases observed in the control and 0.5 mM animals were significantly higher than those observed in the animal of the 5 mM group (One-sample t-test, p:0.0085, and p:0.0047, respectively; (Fig. 6b). The mean percentage area with metastases was 15.18% ± 4.028 in the control animals, and 9.309% ± 2.333 in the 0.5 mM animals, while in the animal of the 5 mM group, only 0.6% of the lung area was affected by metastases.

The mean proliferative index of metastatic neoplastic cells, measured by CDC47 nuclear expression, was significantly lower in the animal of 5 mM group (22.9%, Fig. 6f) than in the control (73.4%, Fig. 6d) and 0.5 mM group (73.5%, Fig. 6e) (One-sample t-test, p:0.0001, and p:0.0012, respectively; Fig. 6c).

### Metformin reduces the expression of EMT and differentiation cells markers in the pulmonary metastasis of murine B16 melanoma

**Figure 7:**
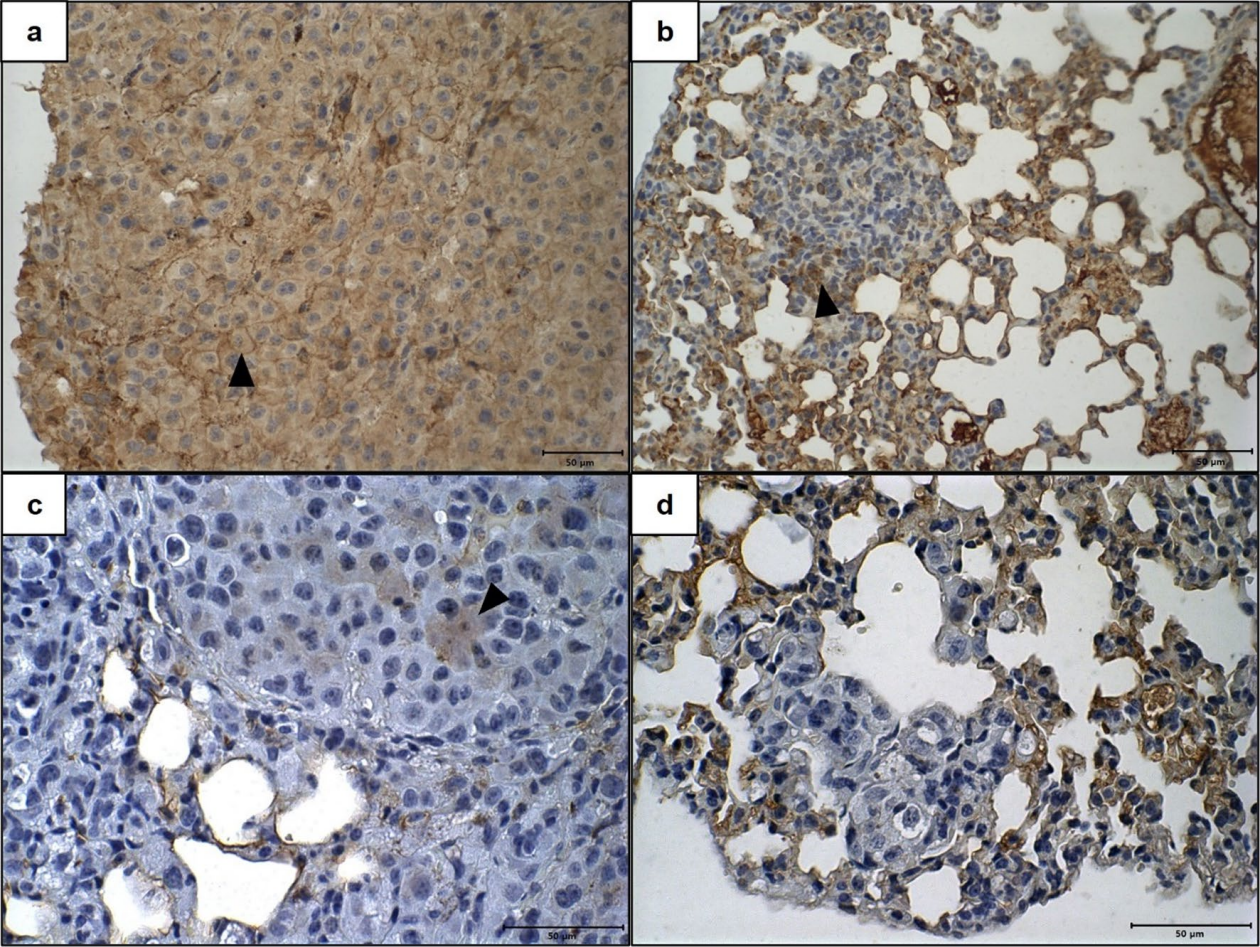
Immunohistochemical expression of N-cadherin and Vimentin in lung metastases from C57BL/6 mice inoculated with metformin-treated or untreated B16-F10 cells. (**a**,**b**) Lung tissues showing membrane and cytoplasmic imunoexpression of N-cadherin 21 days after inoculation of B16-F10 cells: (**a**) untreated with metformin; (**b**) treated, in vitro, with 5 mM of metformin. (**c**,**d**) Lung tissues showing cytoplasmic imunoexpression of vimentin 21 days after inoculation of B16-F10 cells: (**c**) untreated with metformin; (**d**) treated, in vitro, with 5 mM of metformin. ×40. Scale Bar: 50 µM. Arrowhead: Neoplastic cells with positive immunoexpression.

E-cadherin expression was not detected in metastases of any cases, while N-cadherin expression was observed in all cases. N-cadherin expression was observed in cytoplasm and membrane, the score most frequently observed was 3 in the control group (Fig. 7a), in 75% of cases, and score 2 in the 0.5 mM group, in 50% of cases, already in the animals of 5 mM group was observed frequently the score 1 (Fig. 7b). The cytoplasmatic vimentin expression was not detected in metastasis of group 5 mM animal (Fig. 7d), but in the control group 50% of cases present scores 1 (Fig. 7c), and in the 0.5 mM group the most common scores observed was 1 and 2, in 40% of cases each. However, N-cadherin and vimentin expression frequencies do not change between the control and 0.5 mM groups.

**Figure 8:**
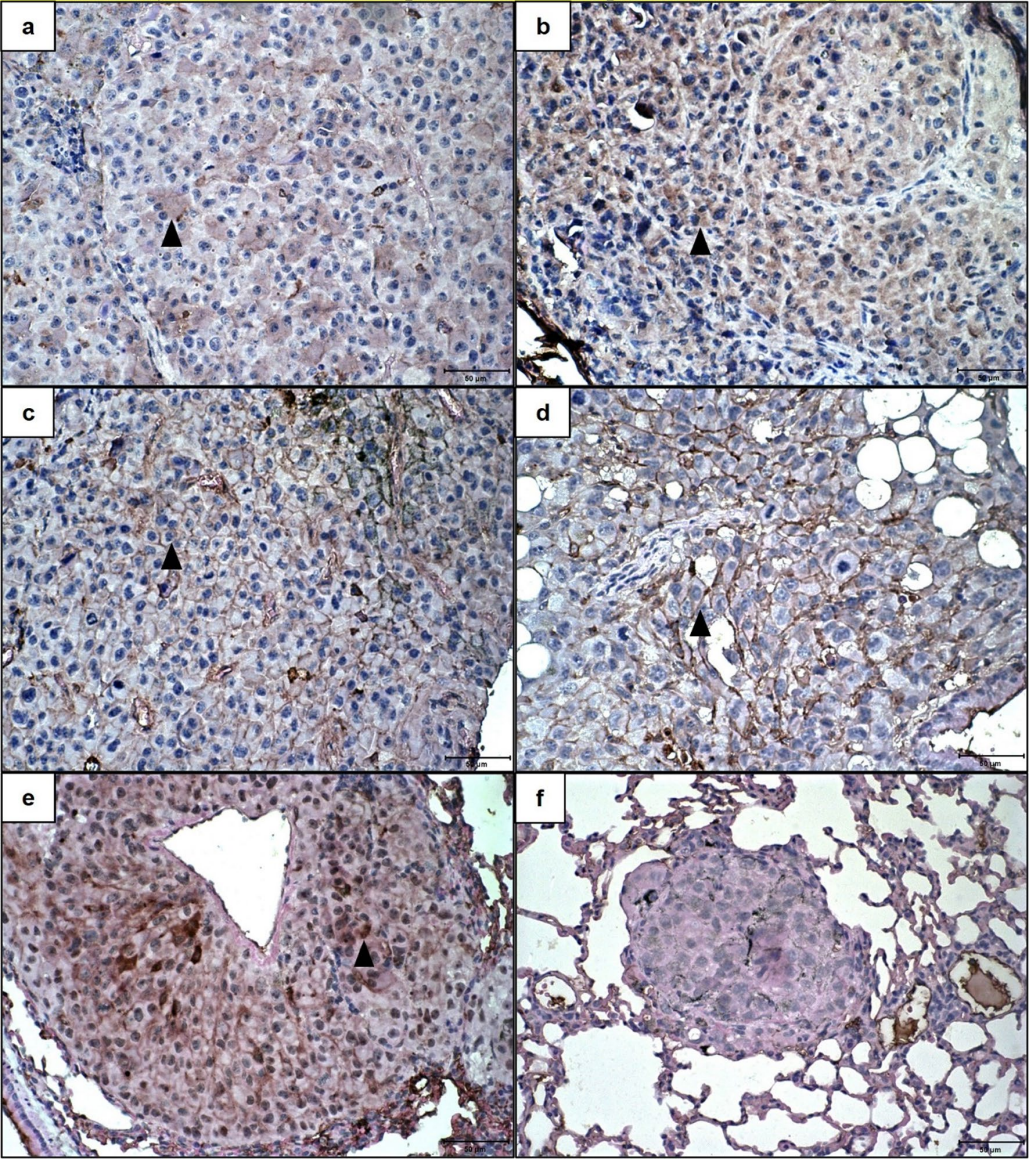
Immunohistochemical expression of Twist, ZEB1, and ZEB2 in lung metastases from C57BL/6 mice inoculated with metformin-treated or untreated B16-F10 cells. (**a**,**b**) Lung tissues showing cytoplasmic imunoexpression of Twist, 21 days after inoculation of B16-F10 cells: (**a**) untreated with metformin; (**b**) treated, in vitro, with 0.5 mM of metformin. (**c**,**d**) Lung tissues showing membrane imunoexpression of ZEB1 21 days after inoculation of B16-F10 cells: (**c**) untreated with metformin; (**d**) treated, in vitro, with 0.5 mM of metformin. (**e**,**f**) Lung tissues showing membrane and nuclear imunoexpression of ZEB2, 21 days after inoculation of B16-F10 cells: (**e**) untreated with metformin; (**f**) treated, in vitro, with 0.5 mM of metformin. ×40. Scale Bar: 50 µM. Arrowhead: Neoplastic cells with positive immunoexpression.

The Twist expression was mainly observed in the cytoplasm, being positive on average at 47.8% and 37.8% of neoplastic cells in the control (Fig. 8a) and 0.5 mM (Fig. 8b) groups respectively. ZEB1 expression, visualized at the membrane, was found only in control (Fig. 8c) and 0.5 mM (Fig. 8d) groups, in an average percentage of 12.5% and 5% of the cells. ZEB2 expression, observed only in the control and 0.5 mM groups, can be seen in the membrane and the nucleus (Fig. 8e,f). Membrane expression was positive on average in 3.5% of cells in the control group and 8.1% in the 0.5 mM group, while nuclear expression was observed in 25% of cells in the control group and 7.5% in the group 0.5 mM, with the expression in the control group being significantly higher than in the 5 mM group (one-sample t-test; p:0.04).

**Figure 9:**
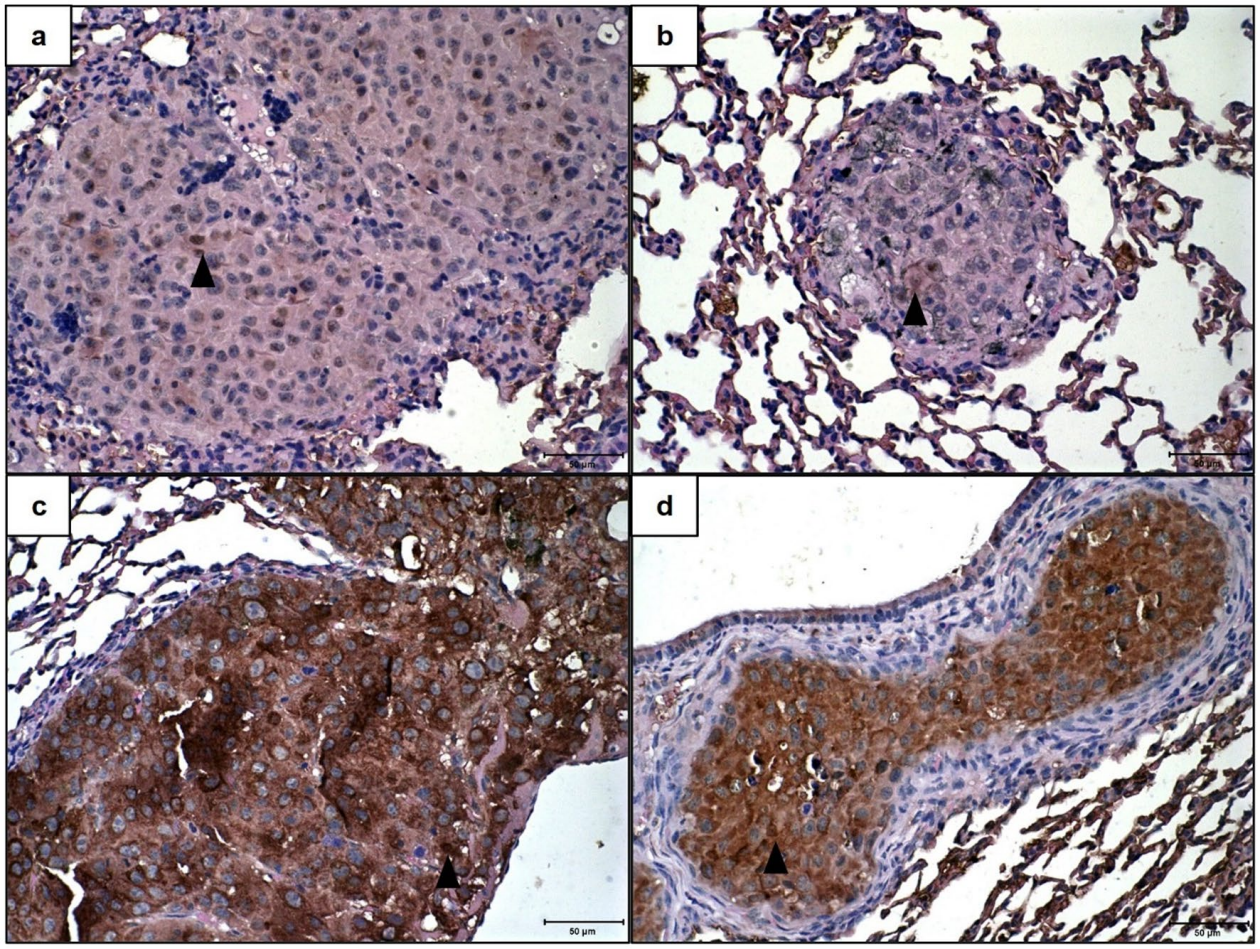
Immunohistochemical expression of Nanog and Sox10 in lung metastases from C57BL/6 mice inoculated with metformin-treated B16-F10 cells. (**a**,**b**) Lung tissues showing nuclear imunoexpression of Nanog 21 days after inoculation of B16-F10 cells: (**a**) untreated with metformin; (**b**) treated, in vitro, with 5 mM of metformin. (**c**,**d**) Lung tissues showing cytoplasmatic imunoexpression of Sox10 21 days after inoculation of B16-F10 cells: (**c**) untreated with metformin; (**d**) treated, in vitro, with 0.5 mM of metformin. ×40. Scale Bar: 50 µM. Arrowhead: Neoplastic cells with positive immunoexpression.

Nanog nuclear expression was observed on average in 14.7% cells/case in the control group (Fig. 9a), 10.2% in the 0.5 mM group, and 8.2% of cells in the animal of 5 mM group (Fig. 9b), but without significative difference between groups.

The intensity of Sox10 expression, observed at the cytoplasm, was predominant moderate (score 2) in 50% of the control group, 62.5% of the 0.5 mM group, and in the group 5 mM animal. The percentage of cells with positive expression of Sox10 was predominantly scored 4 in control (Fig. 9c) (50% of cases) and 0.5 mM group (Fig. 9d) (30% of cases), but in the group, 5 mM animal was scored 1.

## Discussion

Melanoma is a neoplasm of great clinical importance due to high resistance to chemotherapy, fast metastatic spread, and reduced survival time. There is evidence of the occurrence of EMT during its progression.

This study indicated a reduction of in vitro migration in A-375 and B16-F10 cells treated with metformin and in vivo experiments demonstrated that metformin-treated cells generate fewer and smaller metastases. In addition, the metastasis observed in the animal that received 5 mM treated cells showed a reduction in the proliferative index and the expression of mesenchymal (N-cadherin, vimentin, ZEB1, and ZEB2) and cell dedifferentiation marker (Sox10).

Metformin, used in this study to block EMT, affects cellular metabolism^18^, so we performed the MTT assay to rule out toxic concentrations that compromise cell viability and impair the evaluation of EMT blockade. Cytotoxic effects of metformin were observed only in 48-h treatment in both cell lines, consistent with previous studies that show deleterious effects of metformin being more frequent in treatments with higher concentrations, such as 10 mM, or more extended periods (48–72 h)^19,20^, making their use for 24 h viable for our purpose. The A-375 cell line treated with 0.5 mM metformin shows an increasing trend of cell viability but without a statistical difference, this may be an indication that in this concentration metformin increased cell metabolism of A-375 cells, but without significant impacts on cell viability.

The role of EMT in the progression of epithelial neoplasms is widely discussed and well established in the literature, but whether EMT is strictly necessary for the formation of metastases in non-epithelial neoplasms remains to be clarified^7,21,22^. The results obtained in this study show that the reduction of EMT-related markers, promoted by chemical inhibition with metformin, is associated with reducing the potential for metastases formation.

The use of metformin for inhibition of EMT under the conditions applied in this study has already been reported. The mRNA levels were not changed in the in vitro experiments although it was possible to notice differences in the protein level of EMT-related markers when evaluating the expression on pulmonary metastases, showing that the regulation of EMT-related markers in this model, despite not occurring at the genomic level, occurs at the protein level, this may be an indication that the effects of metfomin on EMT can also occur by other mechanisms than direct transcription regulation. The results of the in vivo experiments indicate that the reduction of metastasis formation was concomitant with the reduction of N-cadherin expression. The ability of metformin to inhibit EMT, increase E-cadherin expression, and reduce N-cadherin at the gene and protein level has already been reported in other neoplasms and melanocytic lineages^16,19,23^. This effect has been associated with the inhibitory action of metformin on the MAPK/ERK pathway, which is overactivated in melanomas due to mutations in *BRAF*, *NRAS*, and *NF1* and promotes increased cell proliferation and expression of EMT-inducing transcription factors^24,25^. Recently Suwei et al., show that metformin effects on EMT are dependent on the miR-5100/SPINK5/STAT3 pathway^26^. SPINK5 is a tumor suppressor protein that regulates EMT on melanoma cells, and when overexpressed inhibits metastasis abilities^26,27^. The STAT3 pathway is a known driver stimulator of EMT when phosphorylated at the pY705 site^28^ and SPINK5 could inhibit STAT3 phosphorylation. The treatment with metformin suppressed miR-5100, which targets SPINK5, and elevated SPINK5 expression, reducing STAT3 phosphorylate^26^.

The expression of E-cadherin was not restored in metastasis of metformin-treated cells, however, there is evidence that EMT regulation does not always occur simultaneously on the epithelial and mesenchymal axes^29^.

In melanocytes, N-cadherin expression is associated with a potential for malignant transformation, and in neoplastic melanocytes, it is even considered an indication of a worse prognosis^30,31^. One of the mechanisms by which N-cadherin can facilitate the formation of metastases is by stimulating collective cell migration, where cells maintain physical interconnectivity, collective cell polarity, and coordinated cytoskeletal activity, being a more efficient way than the individual migrating cell^32,33^. One of the main requirements for collective cell migration is remodeling the cell cytoskeleton promoted by Rho-family GTPase signaling, which is stimulated by N-cadherin^34^.

The metastasis in the animal that received 5 mM treated cells shows a reduction in ZEB1 and ZEB2 expression. These proteins are associated with EMT stimulation in several neoplasms, including melanomas^35^. ZEB transcription factors can form a complex with the co-repressors C-terminal binding protein 1 and 2 (CtBP1/2). This complex can bind the CDH1 promoter gene, promoting the E-cadherin downregulating^36^; this effect occurs in epithelial and non-epithelial contexts^10,37^. The pathways directly involved in N-cadherin expression remain unclear^6^, but ZEB2 was one of the few transcription factors that suppress E-cadherin and stimulate N-cadherin expression^38^. On the other hand, some studies show that unlike the observed in epithelial neoplasms, in which ZEB transcription factors act similarly, in melanomas they have antagonistic roles, and the ZEB balance regulation is not yet fully understood^39^. ZEB2 expression is associated with a pro-proliferative status but also cell differentiation, the expression of ZEB1 promotes a mesenchymal/invasive status^10,40^.

Our work shows that the use of metformin for the chemical regulation of EMT-related markers can reduce melanoma's invasive and metastatic potential, which reinforces the importance of EMT in neoplastic progression. Targeting specific molecular pathways associated with epithelial–mesenchymal transition inhibition may provide a more effective strategy in melanoma treatment.

## Materials and methods

### Cell lines and metformin treatment

The melanoma cell lines, A-375 (CRL-1619) and B16-F10 (CRL-6475) were grown, in DMEM supplemented with 10% FBS and 1% penicillin/streptomycin at 37 °C and 5% CO_2_. All cell lines were obtained from American Tissue Culture Collection (ATCC). 24 h prior to the treatment the medium was replaced by DMEM + 5% FBS + 1% penicillin/streptomycin.

Melanoma cell lines were plated in a 96-well plate (5 × 10^3^ cells/well) for MTT assay, or in a 12-well plate (7.5 × 10^4^ cells/well), for RNA isolation. After 24 h the medium was replaced by fresh medium with metformin at concentrations of 0 mM (control), 0.5 mM and 5 mM. Each experiment was repeated at least three times with three replicates per experiment. B16-F10 cells, for in vivo experiments, were cultured at T75 flasks until reaching 70–80% confluence when the metformin was added at concentrations 0, 0.5 mM, or 5 mM for 24 h.

### Analysis of cell viability

Cell viability in A-375 and B16-F10 cells treated with metformin was evaluated using the colorimetric assay MTT. After 24- and 48-h metformin treatment, cells were incubated with 3-(4,5-dimethylthi-azol-2-yl)-2,5-diphenyl-tetrazolium bromide (MTT), for 4 h at 37 °C and 5% CO_2_. Dimethyl sulfoxide was added to lysis cells and released formazan, allowing colorimetric measurement of absorbance in a microplate reader at 570 nm (SpectraMax 190 absorbance microplate reader, Molecular Devices Corp., Sunnyvale, CA).

### RNA isolation and real-time PCR

Total RNA was isolated, after 24 h of metformin treatment, from A-375 and B16-F10 cells using 1 mL of Trizol^®^ reagent according to the protocol provided by the manufacturer (Invitrogen Life Technologies, Carlsbad, CA, USA). First-strand complementary DNA (cDNA) was synthesized from 0.5 μg total RNA using a High-Capacity cDNA Reverse Transcription Kit with RNase Inhibitor (Applied Biosystems, Foster City, CA, USA) incubated in a MiniAmp™ Plus Thermal Cycler (Thermo Fisher Scientific, Wilmington, DE, USA).

Real-time PCR was carried out in QuantStudio 3 Real-Time PCR System (Applied Biosystems, Foster City, CA, USA), using the Power Sybr^®^ Green Master Mix Kit (Invitrogen Life Technologies, Carlsbad, CA, USA). The PCR were performed in three independent experiments, and each sample was run in duplicate in each experiment. Samples were run on 96-well optical PCR plates with a final reaction volume of 20μL. The PCR parameters were 1 cycle at 50 °C for 2 min, 1 cycle at 95 °C for 10 min, 40 cycles at 95 °C for 15 s and 58 °C for 1 min, 1 cycle at 95 °C for 15 s, 60 °C for 1 min and 95 °C for 1 s.

The primers used for PCR amplification of target genes are listed in Table 1. *GAPDH* (glyceraldehyde-3-phosphate dehydrogenase) gene was used as reference to normalize target gene expression. Specific primers were designed using the sequences obtained in GeneBank, through the Blast program (http://www.ncbi.nlm.nih.gov/blast/blast.cgi). Subsequently, all sequences were designed and analyzed using the Integrated DNA Technologies website program (http://www.idtdna.com).

**Table 1:** Primer sequences for qPCR.

Species	Gene	Forward primer (5′–3′)	Reverse primer (5′–3′)
*Homo sapiens sapiens*	*GAPDH*	TGGGTGTGAACCATGAGAAG	GAGTCCTTCCACGATACCAAAG
	*CDH1*	CCCTTCACAGCAGAACTAAC	CACCTCTAAGGCCATCTTTG
	*CDH2*	GGACCGAGAATCACCAAATG	CGTTCCTGTTCCACTCATAG
	*ZEB1*	GGGAGGATGACAGAAAGGAA	GCATCTGACTCGCATTCATC
	*ZEB2*	CCATCTGATCCGCTCTTATC	CCTGTGTCCACTACATTGTC
	*SNAIL*	AGCTGCAGGACTCTAATC	GAGTCCCAGATGAGCATT
	*miR200c*	GTCTTACCCAGCAGTGTTTG	TACCCGGCAGTATTAGAGAC
	*OCT3/4*	GGAGGAAGCTGACAACAATG	CTCACTCGGTTCTCGATACT
*Mus musculus*	*GAPDH*	GTGGAGTCTACTGGTGTCTT	GGTTCACACCCATCACAAAC
	*CDH1*	CATCATTGAGAGGGAGACAG	GACACGGCATGAGAATAGAG
	*CDH2*	CTGACTGAGGAGCCTATGAA	CAGTCTCTCTTCTGCCTTTG
	*ZEB1*	CCAGCAGACCAGACAGTATT	TCTGAGTCACACTCGTTGTC
	*ZEB2*	GCCACGAGAAGAATGAAGAG	CTCCTTGGGTTAGCATTTGG
	*SNAIL*	CAACTATAGCGAGCTGCAGGA	GTACCAGGAGAGAGTCCCAGAT
	*miR200c*	GTCTTACCCAGCAGTGTTTG	TACCCGGCAGTATTAGAGAC
	*OCT3/4*	GATCACTCACATCGCCAATC	CCCTGTAGCCTCATACTCTT

The PCR results were analyzed based on the ΔCT, the primary source of data variability. Relative gene expression was calculated using the 2^−ΔΔCT^ method, where CT is the threshold cycle^41^.

### Cell motility assays

Effects of metformin treatment on cell invasion capacity were evaluated by Corning^®^ Biocoat ™ Matrigel^®^ Invasion Chamber—354480 (Discovery Labware, Inc., Two Oak Park, Bedford, MA, USA), used according to the manufacturer's instructions. A-375 and B16-F10 cells were plated at the seeding density of 2.5 × 10^4^ cells/well into the upper chambers with serum free DMEM + 1% penicillin/streptomycin and metformin at 0, 0.5 or 5 mM concentrations. In the lower chambers was added DMEM supplemented with 5% FBS and 1% penicillin/streptomycin as a chemoattractant. The plate was incubated at 37 °C in 5% CO_2_ for 24 h. The non-invading cells on the upper side of the chamber were removed with a cotton swab and the invading cells that transmigrated to the matrigel and attached on the underside of the chamber were fixed with methanol for 10 min and stained with 0.1% crystal violet for 10 min. Then, the migrated cells were examined using Cytation 5 Cell Imaging Multi-Mode Reader (Biotek, Winooski, VT, USA), and images from 5 random fields/well were captured. Using ImageJ software (version 1.51j8, National Institute of Health, MD, USA), the images were analyzed to determine the number of migrant cells and set to 100% according to the control.

Cell migration after metformin treatment was evaluated by the wound healing assay using the Ibidi Culture-Insert 2 Well system (Ibidi, Martinsried, Germany), a silicon insert that separates two compartments in the wells. The silicone insert was positioned in a 24-well plate and 12.5 × 10^3^ A-375 cells or 7.5 × 10^3^ B16-F10 cells were applied into each compartment with DMEM + 5% FBS and 1% penicillin/streptomycin. After 24 h the insert was removed, cells washed with PBS to remove debris and non-adherent cells, and the adherent cells covered with fresh serum free DMEM + 1% penicillin/streptomycin, 10 µg/mL of mitomycin, and metformin at 0, 0.5 or 5 mM concentrations. Two points of the wound were photographed at 0, 12, 24, 3,0 and 36 h and the area into which the cells migrated was measured using ImageJ software (version 1.51j8, National Institute of Health, MD, USA). The wound closure percentage was calculated according to the formula below^42^.

$${\text{Wound}} \;{\text{closure}} \; ({\%}) =\left[\frac{{A}_{t=0h}-{A}_{t=\Delta h}}{{A}_{t=0h}}\right] \times 100$$

A_t=0 h_: area of the wound at t = 0 h, A_t=Δh_: area of the wound at the interested time.

### Animals

In vivo experiments were performed with C57BL/6 female mice at 5 weeks old, according to the ethical principles for animal experimentation and after approval by the Animal Use Ethics Committee of the Federal University of Minas Gerais (no 143/2019) and complied with the relevant guidelines and regulations. This study is reported in accordance with ARRIVE guidelines. B16-F10 cells 24 h after metformin treatment were mechanically removed, collected, centrifuged, resuspended in sterile phosphate-buffered saline, and quantified in a Neubauer chamber. Mice were inoculated, via tail vein, with 5 × 10^5^ B16-F10 cells (8 animals with no treated cells-control group, 10 animals with 0.5 mM treated cells-0.5 mM group, and 10 animals with 5 mM treated cells-5 mM group). At 21-days post-inoculation, all mice were euthanized with ketamine/xylazine intraperitoneal injection, and lungs were collected for histological analysis.

### Histopathological analysis

Lungs were excised, fixed in 10% neutral buffered formalin (pH 7.4) for 48 h, cleaved, embedded in paraffin, 4 μm-thick sections were obtained, stained with hematoxylin and eosin (H&E), and examined under light microscopy. Neoplastic cells form aggregates, or tumor nodules were considered metastatic lung lesions. The total lung area and area of metastatic lung lesion were measured using images captured at Olympus BX41 microscope in the SPOT 3.4.5 software.

### Immunohistochemistry

Immunohistochemical staining was performed through the peroxidase reaction method with a polymerized secondary antibody and the chromogen 3′3-diaminobenzidine (Novolink Polymer Detection System; Leica Biosystems, Newcastle upon Tyne, UK). Antigen retrieval was performed by incubation in citrate buffer (pH 6.0) at water bath—20 min at 98 °C; or Pascal^®^ Pressure Cooker (Dako Cytomation, Glostrup, DNK)—2 min at 125 °C. Table 2 lists the manufacturers, clones, dilutions, and incubation times for all antibodies used. Tissue slides were counterstained with hematoxylin for 10 s, and Giemsa stain (1:5) for 30 min. Then slides were rinsed with a hydrochloric acid solution (1:100), absolute alcohol, and finally isopropyl alcohol for 1 min Giemsa stain allows the differentiation between melanic pigment, which acquires a greenish hue of chromogen reaction (brownish hue).

**Table 2:** IHC protocol.

Antibody	Manufacturer	Clone	Antigen retrieval	Dilution	Incubation time
E-cadherin	Invitrogen	4A2C7	Water bath	1:100	16 h
N-cadherin	Dako	6G11	Water bath	1:50	16 h
Vimentin	Santa Cruz	Vim3B4	Water bath	1:500	16 h
ZEB1	Sigma-Aldrich	Polyclonal	Water bath	1:400	1 h
ZEB2	Sigma-Aldrich	Polyclonal	Pascal®	1:200	1 h
Nanog	Abcam	Polyclonal	Water bath	1:100	16 h
Twist	Abcam	Polyclonal	Water bath	1:400	16 h
Sox10	Abcam	Polyclonal	Water bath	1:400	16 h
CDC47	Neomarks	47DC141	Water bath	1:400	1 h

### Immunohistochemistry interpretation

Expression of E-cadherin, N-cadherin, and Sox-10 were categorized based on the percentage of positive cells: 0 (negative), 1 (< 25% cells), 2 (25–50% cells), 3 (50–75% cells), and 4 (> 75% cells)^43^. Sox10 expressions were also categorized based on the intensity: 0 (negative), 1 (weak), 2 (moderate), 3 (strong). Vimentin expression was classified in: 0 (negative), 1 (< 5%), 2 (5–25%), 3 (25–50%), 4 (> 50%). Transcription factors (Twist, ZEB1, and ZEB2) labeling was categorized as the percentage of labeled neoplastic cells by a semi-quantitative method. The expression of CDC47 and Nanog have quantitatively evaluated in 500 cells and the percentage of positive cells was determined.

### Statistical analysis

The software GraphPad Prism version 8.0 (GraphPad Software, La Jolla, CA, USA) was used to perform statistical analyses. For quantitative results, means were compared using a one-way ANOVA, one-sample t-test, or t-test and its variants depending on the normality of data distribution. Relationships between qualitative variables were investigated with Fisher’s exact test. The significance level was set to p ≤ 0.05.

## Supplementary Information

Supplementary Legends.

Supplementary Figure S1.

Supplementary Figure S2.

## Data Availability

The datasets generated and used during the current study are available from the corresponding author on reasonable request.
