# Supplementary material for: Epithelial–mesenchymal transition inhibition by metformin reduces melanoma lung metastasis in a murine model
Source: Sci Rep. 2022 Oct 22;12:17776. doi: 10.1038/s41598-022-22235-8 (PMC9588059; doi:10.1038/s41598-022-22235-8)
Supplement: Supplementary file 1 — Supplementary Legends. [file 41598_2022_22235_MOESM1_ESM.docx]

**Supplementary Figure Legends**

**Figure S1. Wound healing assay with A-375 cell line.** Photomicrographs throughout the experimental protocol (0-36 hours) showing the effects of metformin treatment (0.5mM and 5mM) at cell migration to the wound in the cell monolayer.

**Figure S2. Wound healing assay with B16-F10 cell line.** Photomicrographs throughout the experimental protocol (0-36 hours) showing the effects of metformin treatment (0.5mM and 5mM) at cell migration to the wound in the cell monolayer.
