# Supplementary figures and images for: Epithelial–mesenchymal transition inhibition by metformin reduces melanoma lung metastasis in a murine model
Source: Sci Rep. 2022 Oct 22;12:17776. doi: 10.1038/s41598-022-22235-8 (PMC9588059; doi:10.1038/s41598-022-22235-8)

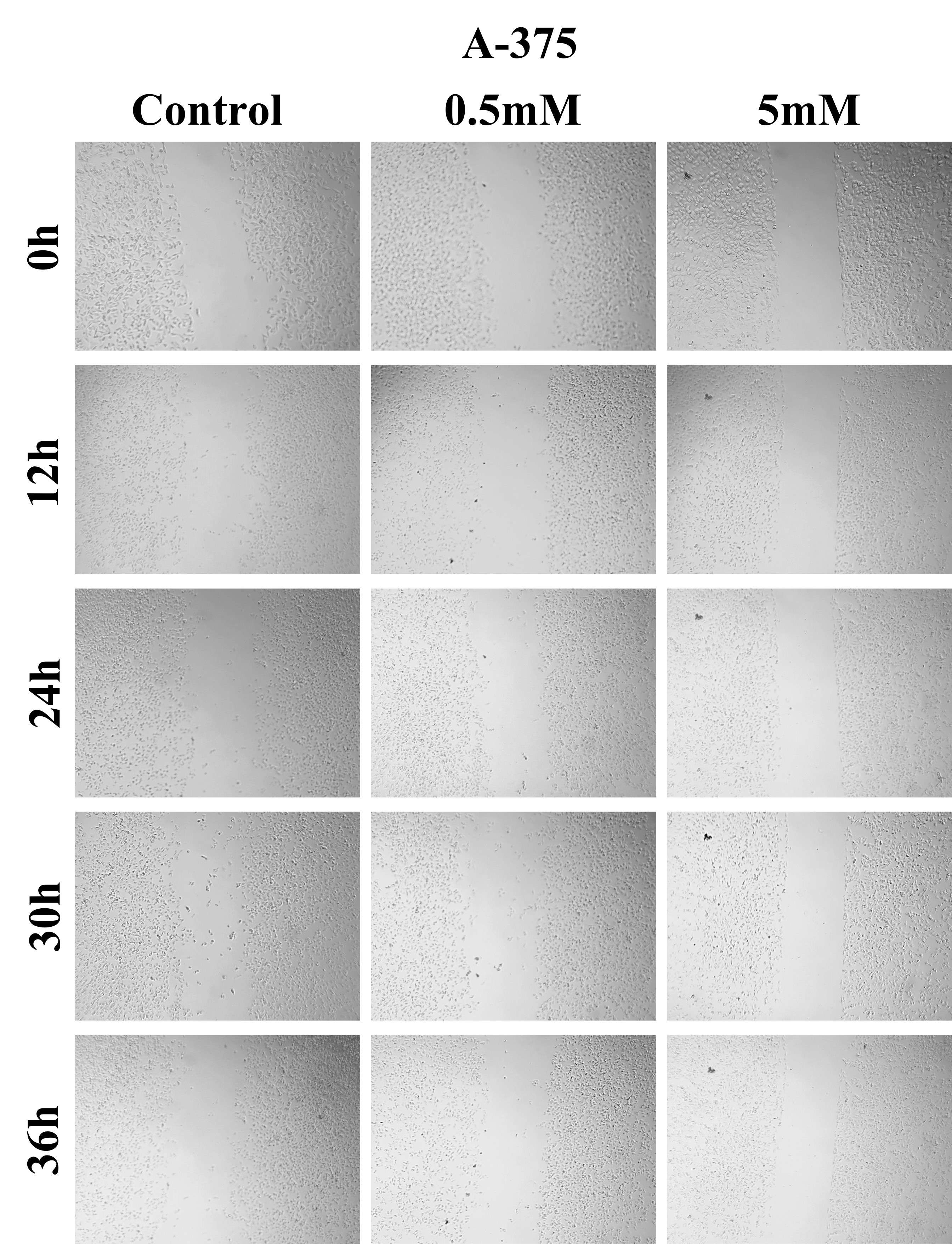

Supplement: Supplementary file 2 — Supplementary Figure S1. [file 41598_2022_22235_MOESM2_ESM.jpg]

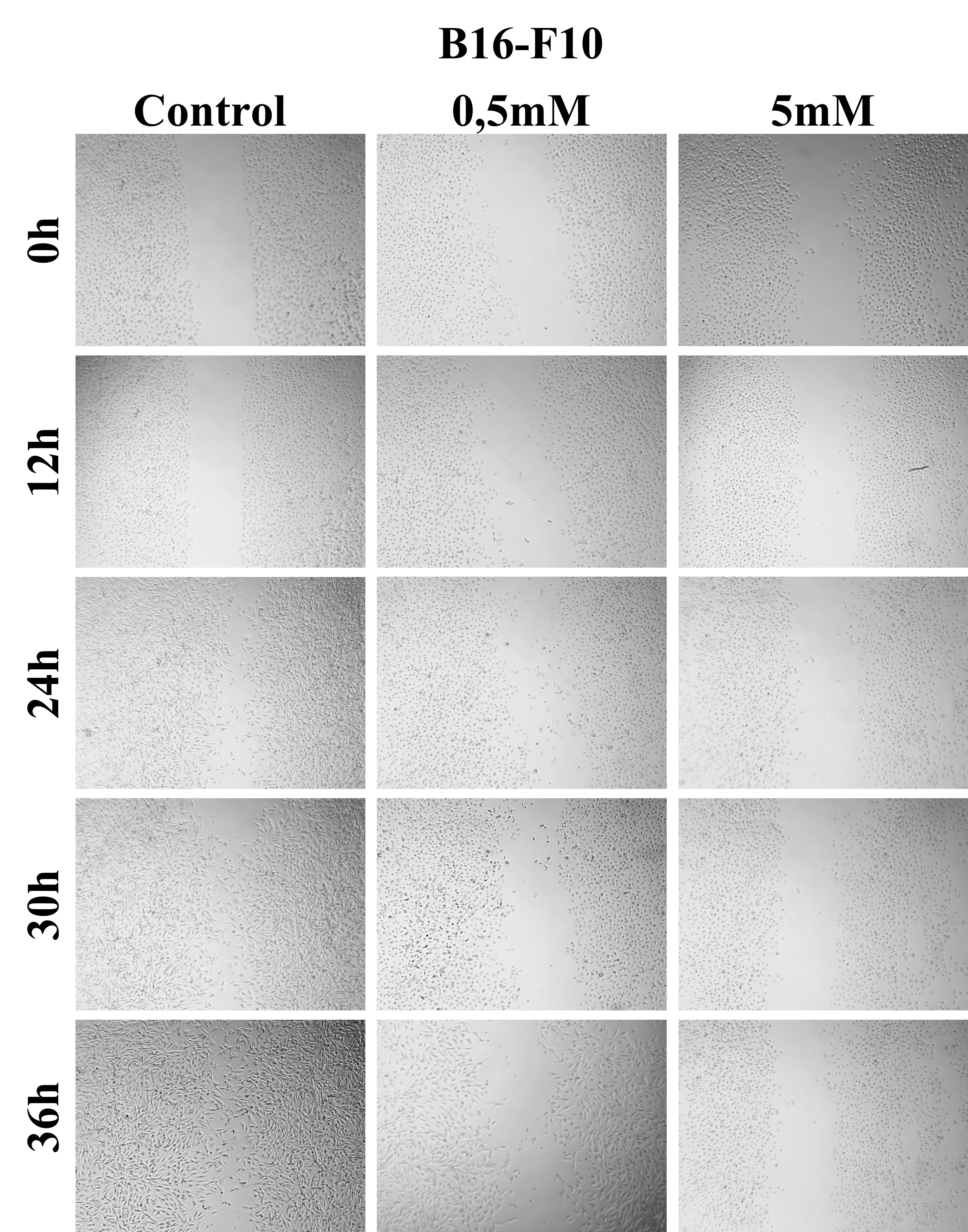

Supplement: Supplementary file 3 — Supplementary Figure S2. [file 41598_2022_22235_MOESM3_ESM.jpg]
